# Giant intrathoracic teratoma presenting with cachexia and severe dyspnea

**DOI:** 10.1186/s13019-019-0922-y

**Published:** 2019-05-22

**Authors:** Emma Ryan, Hani Shennib, Sanjeev Gopal

**Affiliations:** 1 Department of Surgery, University of Arizona, College of Medicine, Phoenix, AZ USA; 2 Mountain Vista Medical Center, 1301 S Crismon Rd, Mesa, AZ 85209 USA; 3 Mesa, USA

**Keywords:** Mediastinum, Lung, Teratoma, Tumor, Resection

## Abstract

**Background:**

This case highlights the challenges of preoperative differential diagnosis and management in a patient with an uncommon clinical presentation of giant intrathoracic teratoma. The age of the patient, location and size of the tumor, and clinical presentation makes this case unique. Typically, intrathoracic teratomas are found between the ages of 20–30, they are located in the anterior mediastinum, and tumors larger than 25 cm clinically present with cough or dysphagia.

**Case presentation:**

A giant intrathoracic teratoma presents in a 51-year-old female as a mid to posterior mediastinal mass compressing the whole left lung with symptoms of depression, anorexia, unintentional weight loss, and cachexia. Due to her severe deconditioning she was optimized for 1 month in a skilled nursing facility with aggressive physical therapy and enteral nutrition. She underwent left thoracotomy with complete resection of the tumor. In follow up her BMI had improved, and she was regaining strength.

**Conclusions:**

Complete resection was achieved via left thoracotomy after aggressive rehabilitation.

## Background

Mature mediastinal teratomas are infrequent, slow growing, and often asymptomatic. Traditionally they present in the fourth decade or earlier and are found in the anterior mediastinum. These teratomas are often found incidentally on imaging and surgical resection can be a challenge due to the size and location of the tumor; however, complete surgical excision is usually curative.

## Case report

A 50-year-old-Caucasian-female with 20-pack smoking years presented with a history of anorexia, lethargy, unintentional weight loss, and depression. Auscultation revealed absent air entry on the left chest. Laboratory workup demonstrated severe iron-deficiency anemia, low albumin, and normal serum alpha-fetoprotein (AFP) and beta-hCG. Pre-operative LDH was not included due to the low suspicion of nonseminomatous dysembryoma or lymphoma.

**Fig. 1:**
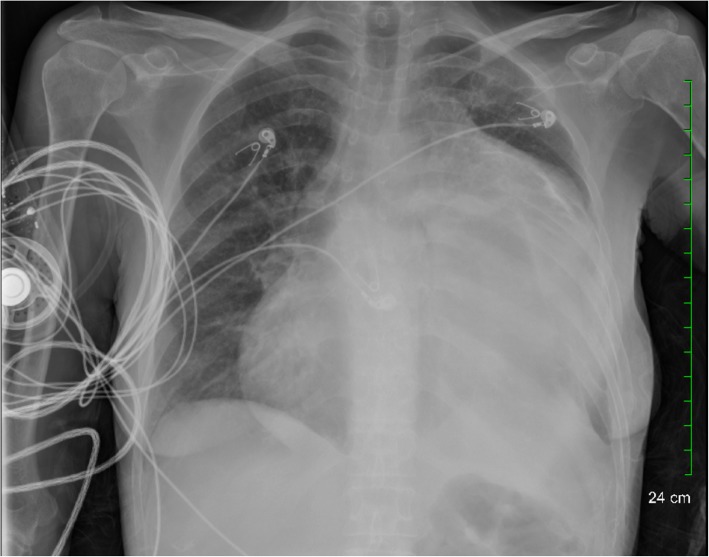
CXR demonstrating a large soft-tissue mass in the left-hemithorax

**Fig. 2:**
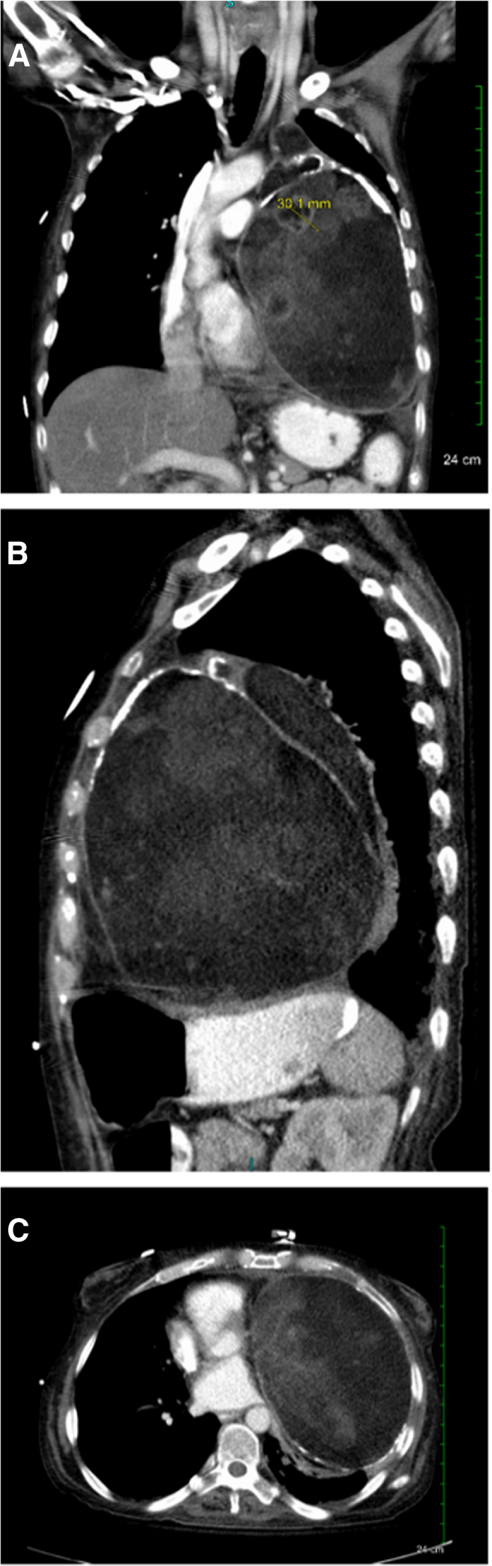
Contrast-enhanced-CT-imaging revealed a 20 cm well-circumscribed dense mass with areas of internal-enhancing-nodularity, creating mass effect on the heart, compressing the left heart and lung hilum. **a** AP **b** Sagittal **c** Transverse

Chest X-ray and computed-tomography revealed a left-thoracic heterogeneously dense 20x25cm mass compressing the heart and lung with a major mediastinal shift (Figs. 1 and 2). A preoperative MRI was not performed as there was no evidence of invasion into any vital structures. Five IR CT-guided core needle biopsies from different regions of the mass yielded minute fragments of amorphous, acellular material which was insufficient for pathological diagnosis. Core needle biopsy has a diagnostic yield of 77% and can be inadequate for immunohistochemistry and flow cytometry evaluation [7]. The profusely dense material ultimately found during the operation largely contributed to the inability of the core needle biopsy to provide a diagnosis.

A preoperative clinical diagnosis of mediastinal teratoma was entertained. Her BMI on admission was 18.4 and she had a Karnofsky performance scale index between 10 and 20. Because of the severe deconditioned status of the patient and cachexia she was put on enteral feeding and sent to a rehabilitation program for 3 weeks. Thereafter the patient underwent left lateral thoracotomy and complete resection of the mediastinal tumor. Lateral thoracotomy was preferred over median sternotomy in this case because the mass did not cross midline and it extended below the level of the pulmonary hilum. A thoracotomy incision is standard approach to a middle or posterior mediastinal mass [4, 5, 7]. Intraoperatively the tumor was firmly adherent to the pulmonary artery and pericardium. It was necessary to resect the anterior aspect of the tumor first to obtain proximal control of the pulmonary artery before completely separating the tumor from the artery.

**Fig. 3:**
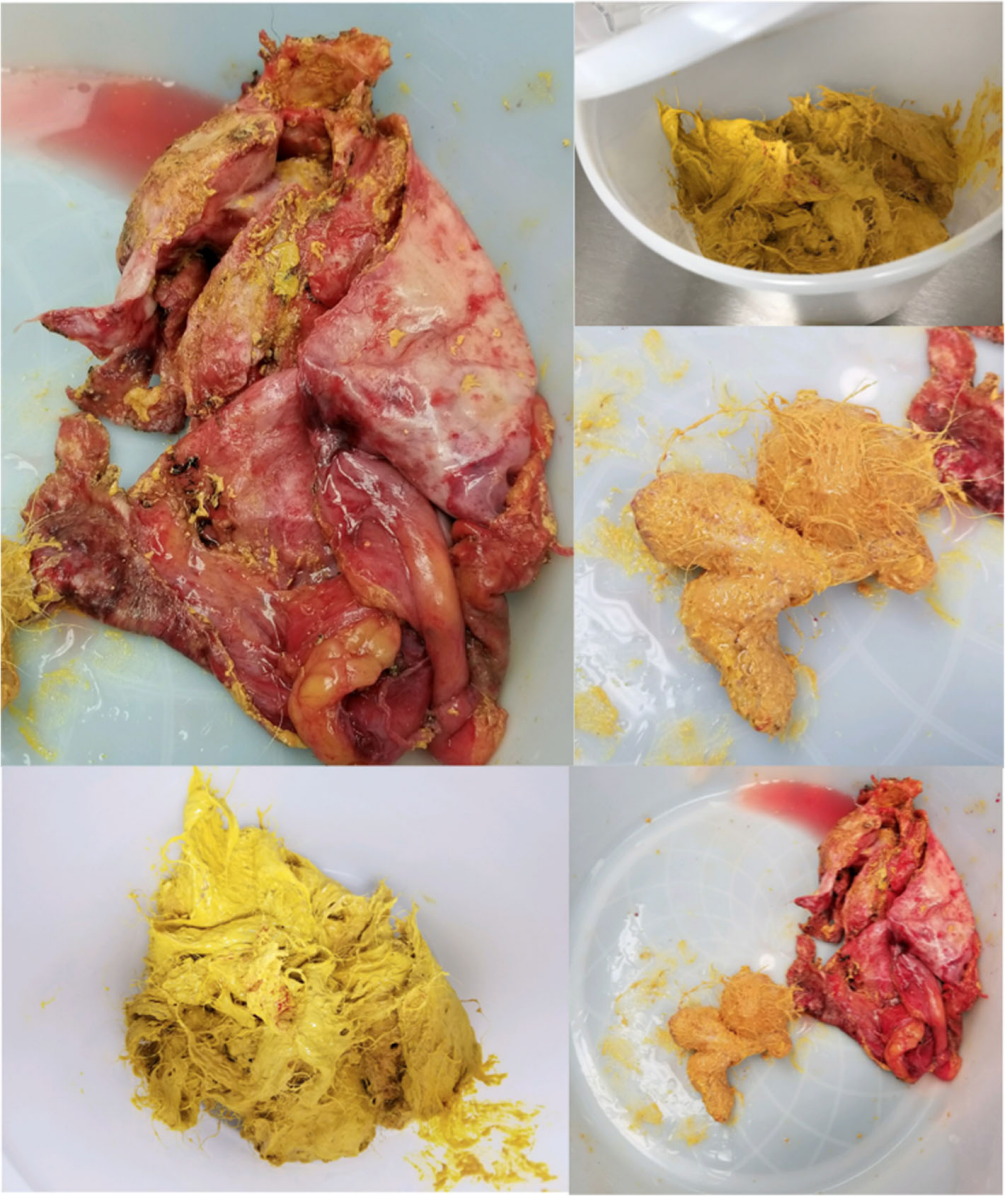
Macroscopic-Mediastinal tumor filled with cheesy, sebum-like material and hair

Macroscopic examination demonstrated yellow pasty-material with teratomatous components including hair and skin (Fig. 3). Histologic description was consistent with mature squamous cells and occasional glandular cells.

The patient required prolonged chest tube drainage to allow for shift of the mediastinum and expansion of the chronically atelectatic lung. She continued physical therapy and enteral feeding supplementation due to severe deconditioning. There were no complications and she was discharged on the 17th postoperative day. At the first follow-up appointment 18 days after discharge she was regaining weight and strength without any clinical evidence of recurrence. Her BMI had increased from 18.4 to 21, and her Karnofsky performance scale index was between 80 and 90, quickly approaching 100. She was scheduled again for follow up in 2 months.

## Comment/discussion

Some rarities of this case require consideration, specifically the older age of our patient, the location and size of the tumor, and the uncommon clinical presentation. While they usually occur equally across gender and more frequently in children and young adults [2], our patient was 51 years old. Ectodermal tissues including skin, hair, and sebaceous glands typically predominate while mesodermal tissues are less commonly seen. These tumors are generally benign, although they can lead to hemorrhage or infection, penetrate the sternum, or rupture into the mediastinum or lung. On gross-examination, they appear as multi-cystic masses containing hair, teeth, and/or skin mixed into sebaceous and often foul-smelling-material. Up to 26% of benign teratomas are calcified with elements of bone or teeth [1, 2].

Thymomas and lymphomas are usually the most frequently occurring neoplasm of the anterior mediastinum while germ cell tumors represent 15% of anterior mediastinal masses. The tumor in our patient was found in the mid to posterior mediastinum. Teratomas are most frequently located within the gonads but can also be found in other regions and have been found in the mediastinum concomitantly [5].

With advanced disease, symptoms are often a result of compression/obstruction of surrounding organs and can include chest discomfort, dyspnea, cough, dysphagia, and rarely expectoration of hair or sebum [1]. Digestive enzymes secreted by gastric, intestinal, or pancreatic tissue within the tumor can have devastating effects on the bronchi, pleura, pericardium, or lung. Neurologic abnormalities can occur such as vocal cord or diaphragmatic paralysis from compression of the recurrent laryngeal nerve or phrenic nerve, respectively.

The work-up for teratomas is predominantly radiographic [6]. Chest x-ray, CT, and MRI can be used to support the preoperative diagnosis and to assess resectability. Benign teratomas are mostly well defined, round, or lobulated anterior mediastinal masses that usually bulge to one side of midline (Fig. 2). A Rokitansky nodule, a solid protuberance projecting from a cyst, often containing calcific dental, adipose, and hair tissue (Fig. 3), can sometimes be identified on imaging. MRI was not obtained in our patient due to low suspicion of invasion of surrounding structures. The identification of fluid, sebaceous elements, fat, and calcifications allows for a prospective diagnosis of mature teratoma, reducing the cost and complications of preoperative tissue diagnosis.

Markers such as AFP and beta-hCG are typically within reference range in benign teratomas but can be produced in high levels by malignant embryonal tumors [1]. Systemic manifestations such as gynecomastia can be caused by beta-hCG; therefore, assaying serum beta-HCG, AFP, and LDH is essential to preventing diagnostic delay of a GCT in patients presenting with extra-gonadal symptoms [2].

Complete surgical excision is almost indefinitely curative for mature mediastinal teratomas. Means of resection include median sternotomy, thoracotomy, or video-assisted thoracoscopic surgery. Subtotal resection can relieve symptoms if complete resection cannot be achieved [5]. Surgical resection can be difficult due to large size or adherence to structures such as the pericardium, lung, great vessels, thymus, sternum, or diaphragm. Although rare, teratomas can have neoplastic potential. Malignant transformation has been reported to occur in 1–2% of ovarian cystic teratomas and in mediastinal teratomas, especially giant mediastinal teratomas [3]. Five-year survival approaches 100%; however, patients must still be followed.

## Conclusion

Again, the age of the patient, location and size of the tumor, and clinical presentation makes this case unique. These tumors are generally benign; however, with advanced disease they can present with compressive symptoms. Radiologic and laboratory workup can support a preoperative diagnosis of teratoma. Surgical excision is frequently curative and, in our patient, complete resection was achieved via left thoracotomy after aggressive rehabilitation.

## References

[1] Duwe BV, Sterman DH, Musani AI (2005). Tumors of the mediastinum. Chest.

[2] Strollo DC, Rosado-de-Christenson ML, Jett JR (1997). Primary mediastinal tumors. Part 1. Tumors of the anterior mediastinum. Chest.

[3] Cantwell BMJ, Richardson PGG, Campbell SJ (1991). Gynecomastia and extragonadal symptoms leading to diagnosis delay of germ cell tumors in young men. Postgrad Med J.

[4] Putnam, Joe. “Chest.” Sabiston Textbook of Surgery; The Biological Basis of Modern Surgical Practice, edited by Courtney M Townsend et al., 20th ed., Elsevier, Inc, 2017, pp. 1607–1612.

[5] Chest Wall, Lung, Mediastinum, and Pleura. Schwartz’s Manual of Surgery, by F. Charles Brunicardi, Dana Anderson, Timothy R. Billiar, David L. Dunn, John G. Hunter, Raphael E, Mcgraw-Hill Medical, 2009.

[6] Tauro LF, Shetty P, Kamath A, Shetty A (2012). Double whammy – mediastinal and ovarian teratoma: a rare clinical co-existence. J Thorac Dis.

[7] Berry, M. Approach to the adult patient with a mediastinal Mass. Uptodate, 13 mar. 2018. www.uptodate.com/contents/approach-to-the-adult-patient-with-a-mediastinal-mass. Accessed 1 Mar 2019.

